# From Cellular Lysis to Microbial Explosion: Elucidating the Temporal Degradation and Spoilage Mechanisms of Frozen Mysid Shrimp as Seahorse (*Hippocampus* spp.) Feed

**DOI:** 10.3390/ani16152325

**Published:** 2026-07-29

**Authors:** Yu Wang, Chenyin Wu, Anna Xu, Siping Li, Shuo Qin, Peng Gao, Hongyu Zhu, Yanming Sui, Tingting Lin

**Affiliations:** 1 East China Sea Fisheries Research Institute, Chinese Academy of Fishery Sciences, Shanghai 200090, China; 18360658619@163.com (Y.W.); 18705100661@163.com (C.W.); lisiping@ecsf.ac.cn (S.L.); 2 College of Marine and Biological Engineering, Yancheng Institute of Technology, Yancheng 224051, China; amyandpancake@163.com (A.X.); qinshuo060215@163.com (S.Q.); fuin6013gopthen@163.com (P.G.); zhy17851995911@outlook.com (H.Z.); suiyanming@ycit.edu.cn (Y.S.)

**Keywords:** frozen mysid shrimp, nutritional degradation, specific spoilage organisms (SSOs), targeted metabolomics, 16S rRNA sequencing

## Abstract

Frozen mysid shrimp is the core feed for commercial seahorse aquaculture, yet quality deterioration caused by long-term cold storage severely constrains the survival and reproductive performance of seahorses. By integrating targeted metabolomics, physicochemical spoilage indicators, and 16S rRNA high-throughput sequencing, this study multidimensionally analyzed the stage-specific mechanisms of nutritional degradation and microecological deterioration of mysid feed at −20 °C under fresh (0 months), short-term frozen (2 months), and long-term frozen (10 months) conditions. The results demonstrate that short-term storage (2 months) effectively suppresses microbial-induced spoilage of mysids; however, freeze–thaw stress triggers cellular lysis, leading to a significant depletion of key water-soluble feeding-attractant amino acids, notably glycine and aspartate. In contrast, long-term frozen storage (10 months) induces severe lipid peroxidation and a massive accumulation of total volatile basic nitrogen (TVB-N). Microbiome analysis confirmed that the fundamental trigger for feed deterioration during the late storage stage is the explosive community succession of psychrotrophic specific spoilage organisms (SSOs), with *Shewanella*, *Photobacterium*, and *Pseudoalteromonas* emerging as the absolute dominant taxa. The highly active extracellular lipases and proteases secreted by these microbial communities extensively degrade the structural lipids of the feed, ultimately resulting in a paradoxical rebound in free fatty acid content during long-term storage. In summary, our research comprehensively details how freezing compromises diet quality through specific biological and chemical pathways. This establishes essential scientific groundwork aimed at refining commercial preservation techniques, executing precise dietary enrichments, and ultimately securing long-term viability across the captive *Hippocampus* breeding sector.

## Simple Summary

Seahorses are increasingly bred in aquaculture to support conservation and meet market demands, heavily relying on frozen mysid shrimp as their primary diet. However, long-term cold storage often deteriorates the feed’s quality, leading to slow growth, elevated mortality, poor gonadal development, and reduced spawning in cultured seahorses. This study investigated how freezing at −20 °C for 2 and 10 months alters the nutritional value, chemical spoilage, and bacterial communities of mysid shrimp. We found that while a short 2-month freeze prevents bacterial spoilage, it causes cells to break open, leading to a significant loss of key feeding-attractant amino acids, such as glycine and aspartate, that stimulate seahorses’ appetite. Alarmingly, extending the freezing time to 10 months leads to severe deterioration. Cold-surviving bacteria multiply rapidly, releasing extracellular lipases and proteases that break down the shrimp’s fats and proteins and generate toxic substances. These findings strongly advise aquaculture facilities to avoid using long-term frozen mysid shrimp and to consider adding nutritional supplements to short-term frozen feed. This approach will help secure a highly nutritious diet for seahorses, supporting the sustainable growth of the artificial breeding industry.

## 1. Introduction

Seahorses (*Hippocampus* spp.) are flagship species for marine biodiversity conservation, listed in Appendix II of the Convention on International Trade in Endangered Species of Wild Fauna and Flora (CITES) and listed as Class II state-protected wild animals in China. Seahorses have been exploited for traditional Chinese medicinal raw materials and ornamental aquarium pets across East Asian coastal regions [1]. Driven by market demand, decades of overfishing have depleted wild seahorse stocks, pushing native seahorse species toward local population depletion [2]. Against severe resource depletion, industrial artificial breeding has gradually evolved into a sustainable alternative to satisfy growing medicinal demands, and it effectively relieves excessive exploitation pressure on wild seahorse germplasm resources [3]. Nevertheless, the large-scale standardized commercial seahorse aquaculture industry still faces multiple unresolved practical bottlenecks, among which unstable breeding survival and reproductive yields serve as the most prominent restrictive factors constraining industrial expansion. Frozen mysid shrimp, as the primary natural prey for seahorses, determines the final survival rate, growth performance, and reproductive efficiency of farmed seahorse populations [4]. In practice, inconsistent nutritional quality of frozen mysid feed triggers slow growth, poor gonadal development, reduced spawning, and elevated mortality in cultured seahorses [4,5].

Low-temperature frozen preservation has become the long-term storage mode for crustacean raw feed materials. Under low-temperature ambient conditions, the catalytic efficiency of endogenous protease, lipase, and other intrinsic digestive enzymes from crustacean tissue is markedly slowed down, thereby delaying rapid feed spoilage to meet the feeding requirements of seahorse cultivation [6,7]. A large volume of nutritional and feed research has confirmed that aquatic raw feeds undergo quality deterioration during long-term low-temperature frozen storage, even under standard conditions. Prolonged frozen preservation causes nutrient loss. Essential amino acids and polyunsaturated fatty acids are degraded via enzymatic hydrolysis and oxidative reactions, while harmful nitrogen-containing and lipid-derived metabolites accumulate during storage [8,9,10]. The quality deterioration of frozen mysids originates from two major categories of factors. First is physical and chemical damage from freezing: Multiple previous frozen shrimp studies reported that ice crystal generation and recrystallization break cell membranes and muscle structures, further triggering protein denaturation and spontaneous lipid oxidation inside feed [11]. After cell structure is destroyed, originally compartmentalized endogenous enzymes leak out and accelerate continuous nutrient decomposition in a cold environment. The second factor comes from microbial activity: existing food microbiology experiments verified that psychrotrophic and facultative anaerobes attached to mysids survive at −20 °C, the conventional freezing temperature for commercial aquatic feed [12]. With extended storage time, microbial community structure changes obviously, and dominant spoilage bacteria decompose protein and lipid to produce harmful metabolites such as histamine and total volatile basic nitrogen (TVB-N), which have been repeatedly observed in cold-stored small crustacean raw material research [13]. These harmful substances reduce feed nutrition and negatively affect the growth and reproduction of cultured seahorses after ingestion [4]. With the application of high-throughput 16S rRNA amplicon sequencing technology, we are able to precisely characterize the dynamic shift of microbial flora during frozen storage [14,15], which further explains the necessity of incorporating microbial diversity detection into our experimental system. According to the above deterioration paths, each of the six testing indicators targets one specific change caused by frozen storage. Long-term freezing degrades protein and fatty acids in mysids; hence, amino acids and free fatty acids are measured to quantify nutritional loss. Microbial metabolism produces nitrogenous toxic products, including histamine and TVB-N, so these two markers are detected to evaluate microbial spoilage. Continuous lipid oxidation generates peroxide products, and thiobarbituric acid reactive substances (TBARS) are used to quantify the oxidation level. Moreover, microbial succession is the core driver of spoilage substance accumulation, which requires 16S rRNA sequencing to clarify the underlying microbial reason. It is necessary to combine all six indicators to fully evaluate overall feed quality variations during cold storage. These core deterioration pathways have been fully verified in farmed penaeid shrimp. Since mysids share similar tissue structure with penaeids, these deterioration rules are also applicable to mysids [16].

Current studies mainly focus on commercial fish and large edible shrimp species. Most existing frozen feed nutrition studies target fish, large shrimp, and terrestrial feeds, whereas few studies focus on frozen mysids [17]. Frozen aquatic feed studies mostly evaluate feed quality through separate single indicators. Many studies independently use TVB-N and histamine to assess the microbial spoilage degree of frozen crustacean feed [18]. TBARS is widely adopted alone to evaluate lipid oxidation deterioration during low-temperature preservation [19]. Previous studies focusing on lipids in polychaetes and free amino acids in deep-water shrimp also adopted this single-indicator detection mode [19,20]. In addition, 16S rRNA high-throughput sequencing has been used to analyze microbial community changes in frozen seafood, but few have applied this technique to frozen mysid feed quality evaluation [21]. Overall, no study has combined these six indicators to systematically assess quality changes of frozen mysids under different storage times. Accordingly, this experiment adopts targeted metabolomics for amino acid and free fatty acid quantification, alongside 16S rRNA high-throughput sequencing to profile microbial diversity. This multi-index combined design aims to reveal the intrinsic correlation between nutrient depletion, physicochemical spoilage, and microbial succession and supply practical data to optimize frozen storage protocols for seahorse mysid feed.

Given the indispensable role of frozen mysid shrimp across the industrial chain of captive seahorse breeding, we propose a research hypothesis: Nutritional divergence caused by varied frozen storage durations is a potential driver of unstable reproductive performance in farmed seahorses. To test this hypothesis, three experimental groups were established in this work: Freshly harvested mysids served as the blank control, while the other two batches were stored continuously at −20 °C for 2 months and 10 months. We determined core nutritional profiles (amino acid and fatty acid composition) and microbial community diversity of mysid specimens; meanwhile, major spoilage biomarkers including histamine, TVB-N, and TBARS were quantified synchronously. Based on these findings, we ultimately propose an optimized regimen that restricts mysid frozen storage to short-term periods coupled with essential nutritional fortification. Implementing this strategy will minimize nutritional loss during preservation, unify quality criteria of frozen mysids, and support steady and sustainable seahorse aquaculture.

## 2. Materials and Methods

### 2.1. Experimental Organisms, Instruments, and Main Reagents

Experimental mysids were collected from brackish-water ponds (salinity: 12‰) in Haiwan Town, Fengxian District, Shanghai, China. Sampling was conducted at night. Approximately 6 kg of live mysids was harvested by fishermen using trawl nets and transported overnight to the Ninghai Experimental Base of the East China Sea Fisheries Research Institute in Zhejiang Province via live seafood transport vehicles. Upon arrival at the experimental base, mysids were collected and drained immediately and then rinsed repeatedly with boiled and cooled seawater. Afterwards, samples were subpackaged into small containers (approximately 1 kg per container) at a mysid-to-seawater weight ratio of 7:3 (*w*/*w*) and preserved frozen at −20 °C in a freezer for subsequent use. Based on a previously reported cold storage scheme for feed mysids [4], three storage treatments were established: untreated fresh live mysids, and mysids hermetically stored at −18 °C for either 2 or 10 months. For six detection indices including free amino acids, free fatty acids, TBARS, TVB-N, histamine, and 16S rRNA microbial sequencing, the following instruments and chromatographic reagents were used.

An ultra-performance liquid chromatography-tandem mass spectrometry (UPLC-MS/MS) system (AB SCIEX, Framingham, MA, USA) quantified free amino acids and free fatty acids. A thermostatted shaking water bath, a refrigerated high-speed centrifuge, an ultrasonic cleaner, and a nitrogen evaporator were used for all sample pretreatment processes. The Kjeltec 8400 distillation device (FOSS, Hillerød, Denmark) was applied for TVB-N determination. A UV–Vis spectrophotometer was adopted to measure the TBARS value. A fluorescence high-performance liquid chromatograph was used for histamine detection. PCR amplifier (Bio-Rad Laboratories, Hercules, CA, USA) and Illumina MiSeq sequencing platform (Illumina, San Diego, CA, USA) were prepared for 16S rRNA gene sequencing analysis. Chromatographically pure methanol, acetonitrile and formic acid, and mixed standard solutions of amino acids and free fatty acids were purchased from commercial suppliers.

### 2.2. Determination of Amino Acids

To prepare the biological samples, approximately 20 mg of tissue was mechanically disrupted in a cold 80% methanol aqueous solution. The suspension was sonicated on ice for 20 min and subsequently stored at −20 °C for 60 min. The mixture was then centrifuged at 16,000× *g* for 20 min at 4 °C. The supernatant was collected and evaporated to dryness. The resulting dried residue was redissolved in refrigerated 50% methanol. Prior to instrumental analysis, a final clarification was performed by centrifugation at 20,000× *g* for 15 min at 4 °C.

Based on the method [20], a specialized mass spectrometry workflow was employed to quantify free amino acids. Chromatographic separation was achieved using two mobile phases: Mobile phase A consisted of 10 mM ammonium formate and 0.1% formic acid in water, while mobile phase B was pure acetonitrile. The column temperature was maintained at 40 °C, the flow rate was set to 300 μL/min, and the injection volume was 2 μL. The gradient elution program was as follows: The initial organic phase (B) was held at 90% for 1 min, linearly decreased to 40% over the next 8 min, maintained at 40% until 10.5 min, rapidly increased back to 90% within 6 s, and held at 90% until the end of the 12 min run. Mass spectrometric analysis was conducted using positive electrospray ionization (ESI+) in multiple reaction monitoring (MRM) mode. The instrument source parameters were set as follows: source temperature of 550 °C, nebulizer gas at 40 psi, auxiliary heating gas at 50 psi, curtain gas at 35 psi, and an ion spray voltage of 5500 V. Finally, data evaluation was performed using MultiQuant software (version 3.0.2), and target metabolite concentrations were calculated based on established calibration curves.

### 2.3. Determination of Free Fatty Acids

Biological specimens were defrosted inside a 4 °C environment. Exactly 50 mg of this softened material was combined alongside an internal reference spike plus the classic Dole extraction formulation (comprising forty parts isopropyl alcohol, ten parts heptane, and one part sulfuric acid by volume). We allowed these combined constituents to incubate without external heating for ten minutes, promoting complete chemical interaction. Following multiphase partitioning, researchers spun the resulting liquids at 20,000× *g*. This rotation lasted fifteen minutes within a controlled twenty-degree Celsius centrifuge. Technicians carefully gathered the lighter, non-aqueous fraction, subsequently removing all solvents using a continuous gaseous nitrogen stream. Adhering to marine fat profiling methodologies established by Standal and co-workers [19], dried residues underwent a consecutive chemical tagging process. This involved initial treatment utilizing oxalyl chloride, followed directly by 3-picolylamine application. Finally, investigators reconstituted these newly tagged compounds using pure methanolic solvents before introducing them into the analytical equipment.

The separation workflow relied upon two customized carrier fluids. Eluent A comprised water containing zero-point-one percent formic acid, whereas Eluent B incorporated identical acid concentrations dispersed throughout a mixture containing seventy percent acetonitrile alongside thirty percent isopropyl alcohol. System hardware maintained column heating near forty degrees Celsius, pushing liquids steadily at 300 μL/min, while introducing precise 5 μL aliquots per cycle. The programmed delivery shifted dynamically: Initial pure aqueous flow (0–1 min) transitioned steadily toward complete organic saturation by minute eight. This maximum organic state persisted through minute fourteen, before rapidly reverting toward baseline aqueous conditions terminating at sixteen minutes. Spectrometric hardware configurations mirrored the exact operating parameters previously implemented during unbound amine evaluations. The analytical platform operated exclusively by employing multiple reaction monitoring techniques, allowing scientists to determine exact unbound lipid concentrations through mathematical interpolation against pre-validated calibration plots.

### 2.4. Determination of Thiobarbituric Acid Reactive Substances (TBARSs)

To assess the severity of lipid peroxidation, the generation of thiobarbituric acid reactive substances (TBARSs) was measured according to the Chinese national standard GB 5009.181 [22]. Briefly, 10 g of the homogenized sample was mixed with 50 mL of an extraction solution containing 7.5% trichloroacetic acid and 0.1% EDTA. The mixture was continuously shaken in a water bath at 50 °C for 30 min. After cooling to room temperature, the homogenate was filtered through double-layer filter paper. Subsequently, 5 mL of the filtrate was mixed with 5 mL of 0.02 M 2-thiobarbituric acid solution. The mixture was incubated in a water bath at 90 °C for 30 min and then cooled to room temperature. Absorbance was measured at 532 nm. The TBARS value was quantified using 1,1,3,3-tetraethoxypropane to construct a standard calibration curve, and the results were expressed as milligrams of malondialdehyde per kilogram of sample (mg MDA/kg).

### 2.5. Determination of Total Volatile Basic Nitrogen (TVB-N)

The total volatile basic nitrogen (TVB-N) content was determined according to the Chinese national standard GB 5009.228 [23]. Briefly, 10 g of the minced sample was mixed with 75 mL of purified water, and the suspension was shaken for 30 min to ensure adequate extraction. Subsequently, 1 g of magnesium oxide (MgO) was added to the homogenate, and the mixture was immediately transferred to a Kjeltec 8400 distillation unit (Foss, Denmark). The released volatile nitrogen was distilled and collected in a receiving flask containing a 2% boric acid aqueous solution mixed with a dual indicator (bromocresol green and methyl red). Finally, the collected distillate was titrated with 0.1 M HCl to the designated visual endpoint to calculate the TVB-N concentration.

### 2.6. Determination of Histamine

Histamine content was determined using high-performance liquid chromatography (HPLC) (Agilent Technologies, Santa Clara, CA, USA) after pre-column derivatization with o-phthalaldehyde (OPA), according to the method described by Zhai et al. [24]. Briefly, 5 g of the tissue sample was homogenized in 30 mL of 10% trichloroacetic acid (TCA). The homogenate was centrifuged at 1000× *g* for 15 min at 4 °C. The supernatant was collected, and the solid residue was re-extracted with an additional 10 mL of 10% TCA, followed by centrifugation under the same conditions. The resulting supernatants were pooled, filtered, and stored at 4 °C prior to derivatization.

To prevent light degradation, the subsequent derivatization reaction was performed in the dark. Exactly 150 μL of the extract was mixed with 1.9 mL of ultrapure water and 0.4 mL of 1 M NaOH. Immediately afterward, 100 μL of o-phthalaldehyde (OPA) solution (10 mg/mL in methanol) was added. The mixture was thoroughly vortexed and incubated for 4 min. Finally, the reaction was terminated by adding 0.2 mL of 3 M HCl.

A 20 μL aliquot was injected into the HPLC system. Chromatographic separation was achieved using a Hibar Lichrosorb RP-18 column (Merck KGaA, Darmstadt, Germany) (125 mm × 4 mm i.d., 5 μm). Fluorescence detection was performed with excitation and emission wavelengths set at 358 nm and 447 nm, respectively. Isocratic elution was applied using a mobile phase consisting of acetonitrile and 25 mM potassium dihydrogen phosphate buffer (1:1, *v*/*v*) at a flow rate of 0.7 mL/min. The retention time of the target analyte was approximately 4.56 min. All extractions were performed in duplicate, and each extract was analyzed in triplicate.

### 2.7. 16S rRNA Gene Sequencing

To isolate complete microbiological genomes from the animal tissues, researchers employed commercial silica-membrane extraction assemblies (Tiangen Biotech, Beijing, China), strictly adhering to factory-provided instructions. Following isolation, analysts evaluated nucleic acid yields alongside purity parameters before proceeding toward targeted gene amplification.

Specific sequences within the 16S ribosomal RNA region were multiplied via specialized oligonucleotides. We utilized forward sequence A16SF (5′-GGGAGTCCTTCGGGAATCAGA-3′) paired alongside its complementary reverse counterpart—a combination frequently adopted across historical marine arthropod microbiome investigations [25]. The twenty-five-microliter assay mixture comprised template material, primer sets, RNase/DNase-free liquid, and twelve-point-five microliters of double-strength My Taq HS Red Mix. Thermal cycling occurred via conventional laboratory methodologies.

Upon confirming amplicon suitability, these resultant molecules served as foundations for building sequencing libraries. Next-generation genetic analysis ran entirely atop an Illumina MiSeq instrument architecture. Finally, researchers uploaded all unmodified read collections into the public NCBI BioProject repository, making them globally accessible utilizing identifier code PRJNA1475597.

### 2.8. Statistical Analysis

Quantitative results appear formatted, reflecting averages plus or minus standard deviation metrics. To execute baseline mathematical assessments, investigators utilized IBM SPSS Statistics (version 27.0). Determining significance across multiple experimental cohorts involved conducting one-way analysis of variance procedures. Whenever researchers detected initial variations, they subsequently applied Duncan’s post hoc comparison methods, defining meaningful differences only when probability values fell beneath the 0.05 threshold. Conversely, handling complex genomic datasets required distinct computational tools. The 4.2.1 release of R environments, augmented by relevant specialized libraries, managed all transcriptomic information interpretation alongside advanced chart generation.

## 3. Results

### 3.1. Changes in Nutritional Quality During Frozen Storage

#### 3.1.1. Dynamics of Free Amino Acids Profiles

Relative to fresh mysids, the majority of essential amino acids—specifically leucine, valine, histidine, phenylalanine, threonine, and isoleucine—were significantly depleted in both frozen storage groups (Table 1). Fresh samples harbored an exceptionally rich pool of pleasant free amino acids (PFAAs), which was strictly dominated by glycine (16,292.86 μg/g) and aspartate (5337.56 μg/g). However, following 2 and 10 months of freezing, most amino acids experienced a sharp reduction, with concentrations typically plummeting by over 50% to 70%.

When comparing the two frozen stages, the vast majority of PFAAs—including glycine, threonine, glutamate, aspartate, alanine, proline, and arginine—showed no significant intergroup differences between month 2 and month 10 (*p* > 0.05). The sole exception was serine, which continued to degrade significantly from 898.59 μg/g at 2 months to 480.70 μg/g at 10 months (*p* < 0.05).

A highly consistent pattern was observed among unpleasant free amino acids (UPFAAs). Six out of the nine identified UPFAAs (leucine, lysine, valine, methionine, histidine, and cysteine) remained relatively stable across the two freezing durations. Conversely, the remaining three (isoleucine, phenylalanine, and tyrosine) underwent further significant degradation, yielding markedly lower levels at 10 months compared to 2 months (*p* < 0.05).

Regarding amino acids with physiological regulatory functions, glutamate, lysine, and arginine displayed no significant variations between the two frozen groups (*p* > 0.05). Notably, glutamine exhibited a distinct and significant decline during the prolonged freezing phase, falling from its peak of 663.60 μg/g at month 2 to 528.88 μg/g at month 10 (*p* < 0.05). Overall, despite the continued decomposition of specific metabolites, the global free amino acid profile remained largely stable between the 2-month and 10-month frozen treatments, lacking widespread significant differences.

#### 3.1.2. Alterations in Free Fatty Acids

Cryopreservation at −20 °C exerted a significant impact on the free fatty acid (FFA) content of mysids (Table 2). Compared with the fresh group, cryopreservation led to a significant decrease in the levels of several key fatty acids, including oleic acid (9-Octadecenoic acid, C18:1), eicosapentaenoic acid (EPA, C20:5), and arachidonic acid (ARA, C20:4).

When comparing the two frozen storage durations, the evolution of FFAs exhibited non-linear dynamic characteristics. Unexpectedly, the absolute content of FFAs did not simply decrease with longer storage times. Conversely, the contents of numerous key fatty acids in the 10-month frozen group were significantly higher than those in the 2-month frozen group. For instance, the ARA content dropped to its nadir in the 2-month frozen group (26.73 nmol/g) but showed a significant rebound in the 10-month group (43.95 nmol/g, *p* < 0.05). A similar recovery trend was also observed for several other high-value indicators, such as myristic acid (C14:0), palmitoleic acid (C16:1), and linoleic acid (C18:2).

### 3.2. Accumulation of Spoilage Biomarkers and Lipid Oxidation

#### 3.2.1. Degree of Lipid Oxidation: TBARS Analysis

**Figure 1:**
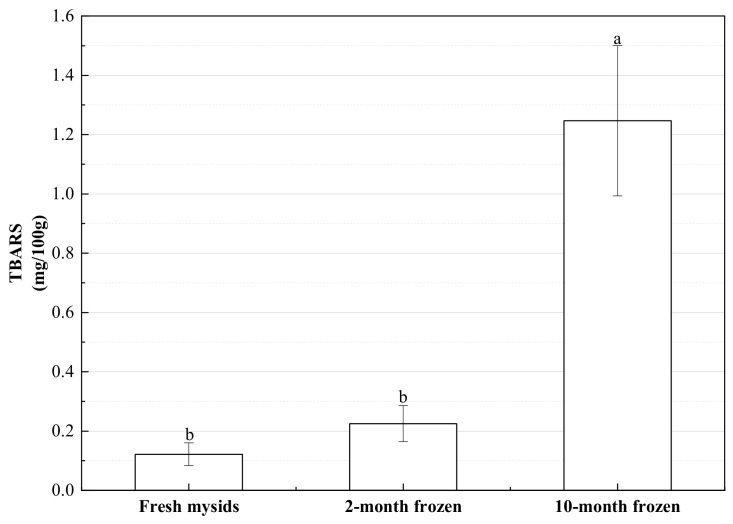
Thiobarbituric acid reactive substances (TBARS) concentrations in fresh mysids, mysids frozen for 2 months, and mysids frozen for 10 months. Different lowercase letters above the bars indicate significant differences among the storage periods (*p* < 0.05, Duncan’s post hoc test).

The continuous generation of peroxide products was assessed via TBARS. As illustrated in Figure 1, fresh mysids maintained a low baseline TBARS value of 0.122 mg/100 g, which increased moderately, though not significantly, to 0.225 mg/100 g after two months. Strikingly, extending the storage to 10 months triggered a sharp and significant elevation, reaching a maximum of 1.247 mg/100 g (*p* < 0.01). This indicates severe spontaneous lipid autoxidation and enzymatic breakdown during the later stages of cold storage.

#### 3.2.2. Protein Degradation and Toxic Metabolite Formation: TVB-N and Histamine

**Figure 2:**
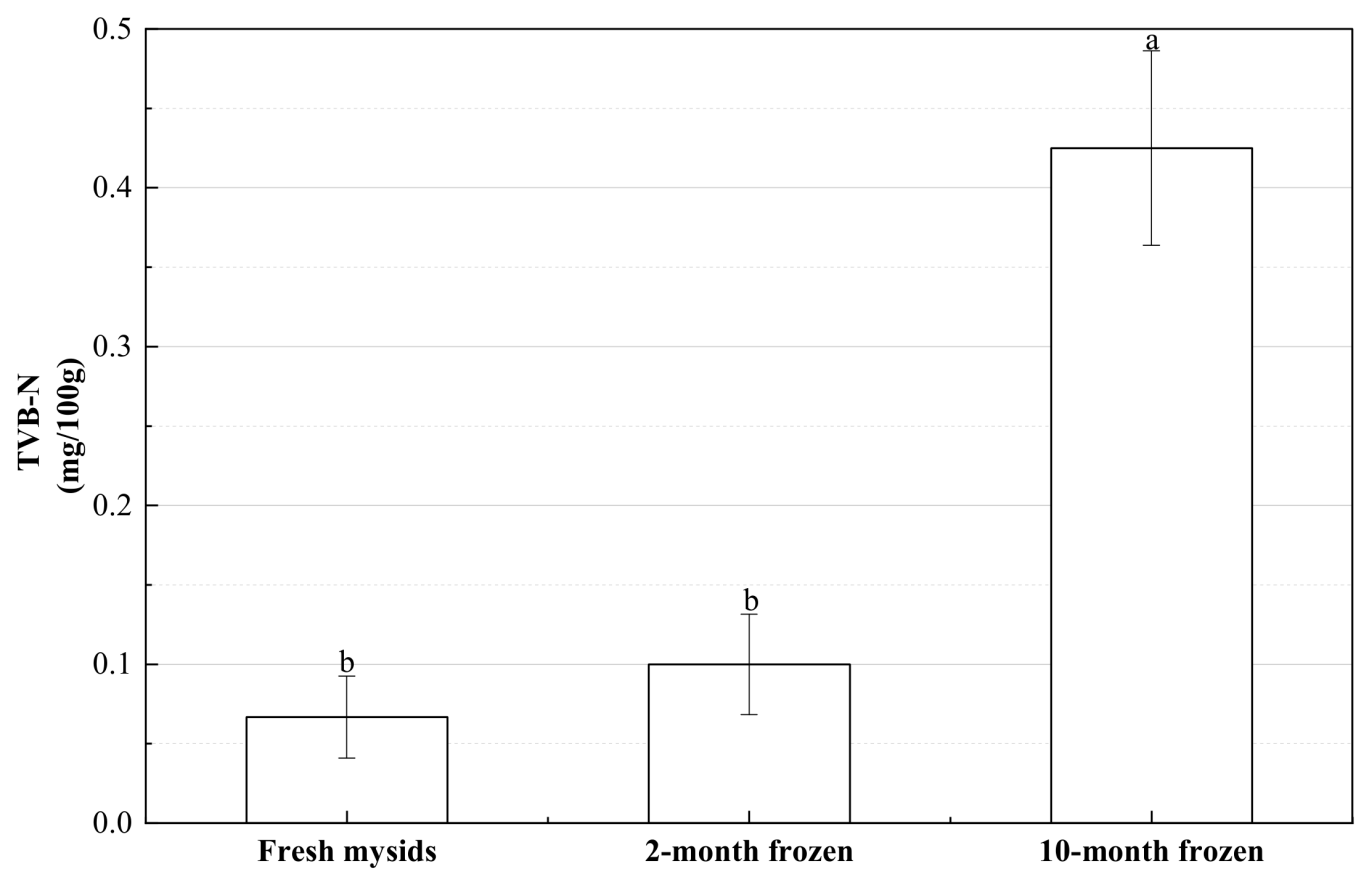
Total volatile basic nitrogen (TVB-N) contents in fresh mysids, mysids frozen for 2 months, and mysids frozen for 10 months. Different lowercase letters above the bars indicate significant differences among the storage periods (*p* < 0.05, Duncan’s post hoc test).

**Figure 3:**
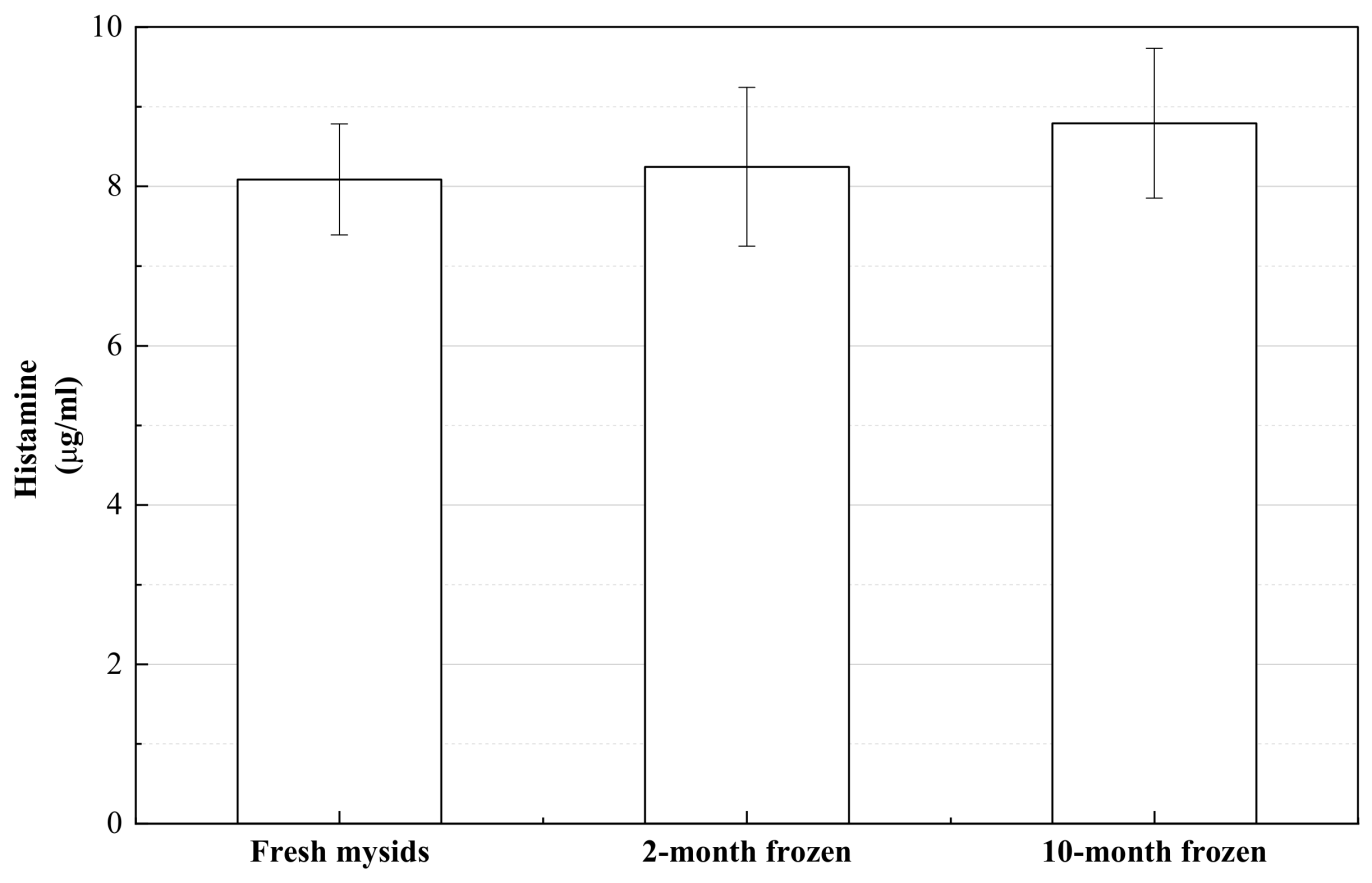
Histamine concentrations in fresh mysids, mysids frozen for 2 months, and mysids frozen for 10 months.

Mirroring the trend of lipid oxidation, the total volatile basic nitrogen (TVB-N) content surged predominantly in the late storage phase (Figure 2). The TVB-N values between fresh (0.067 mg/100 g) and 2-month frozen samples (0.100 mg/100 g) showed no significant difference. However, the 10-month treatment exhibited a dramatic accumulation of TVB-N, peaking at 0.425 mg/100 g (*p* < 0.01). In contrast, histamine concentrations remained overall stable across all treatment groups, ranging from 8.0875 μg/mL in fresh samples to 8.7912 μg/mL after 10 months of storage, with no significant differences among the groups (Figure 3).

### 3.3. Succession of Microbial Community Driven by Storage Time

#### 3.3.1. Shifts in Taxonomic Composition and Dominant Spoilage Bacteria

**Figure 4:**
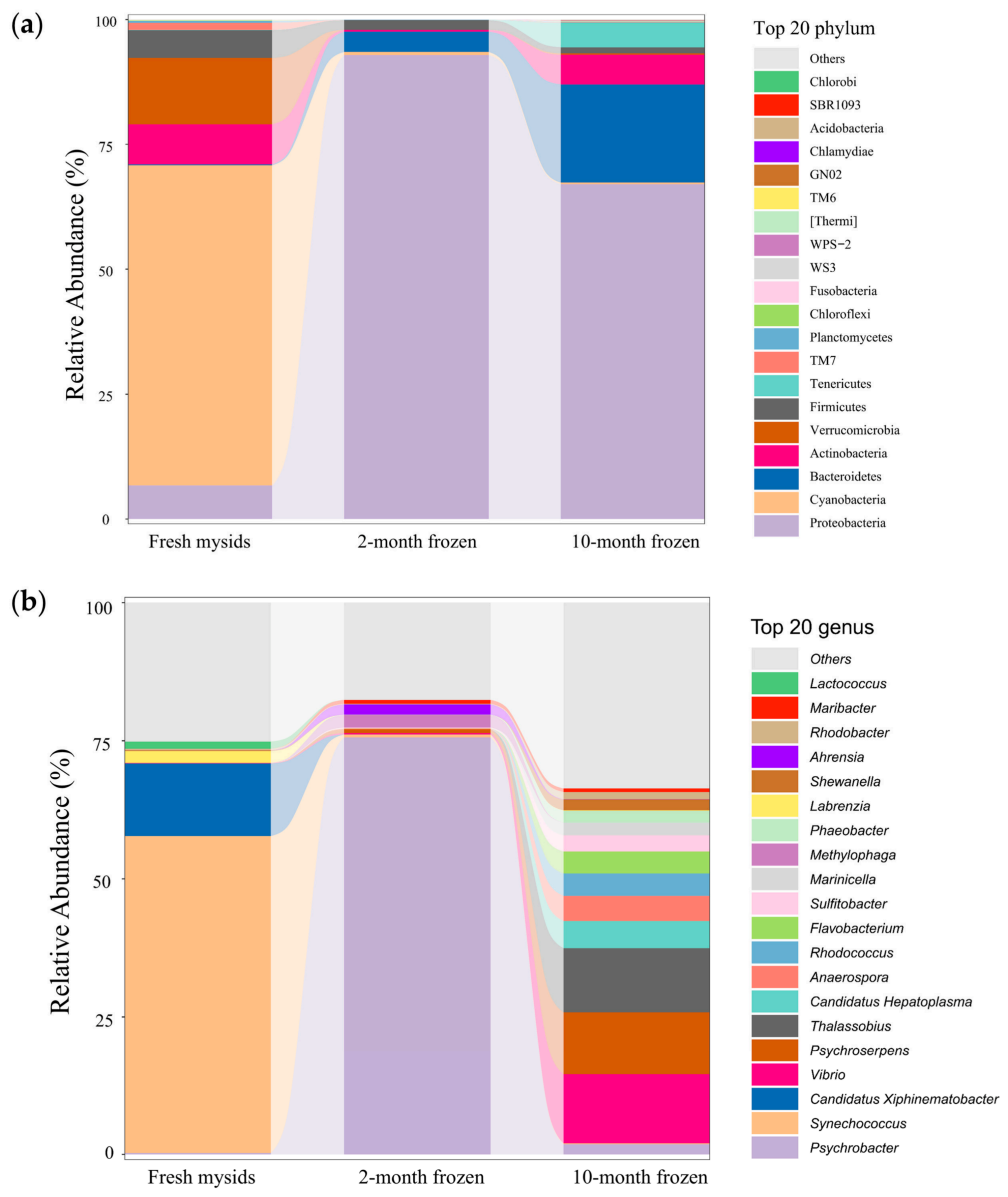
Relative abundance of microbial communities in fresh and frozen mysids. (**a**) Changes in the relative abundance of the top 20 microbial phyla across fresh, 2-month frozen, and 10-month frozen samples. (**b**) Changes in the relative abundance of the top 20 microbial genera across fresh, 2-month frozen, and 10-month frozen samples.

The results of 16S rRNA gene amplicon sequencing revealed that the freezing treatment significantly altered the microbial community structure of the mysids, demonstrating distinct time-dependent successional patterns. At the phylum level (Figure 4a), the microbial community in the fresh mysid samples was strongly dominated by Cyanobacteria, accompanied by a certain proportion of Actinobacteria. However, after 2 months of frozen storage, the community structure underwent drastic changes; the original dominant phyla almost completely disappeared, while Proteobacteria proliferated rapidly to become the sole absolute dominant phylum. As the freezing period extended to 10 months, although Proteobacteria remained the most abundant group, its relative abundance decreased significantly compared to the 2-month group. Concurrently, community diversity increased, with Bacteroidetes emerging abundantly to occupy a substantial proportion. The abundance of Actinobacteria also partially recovered, accompanied by the emergence of Firmicutes.

Analysis at the genus level (Figure 4b) further elucidated these successional dynamics. The fresh samples were primarily dominated by *Synechococcus*. After 2 months of freezing, *Psychrobacter*, a genus highly adapted to low-temperature environments, completely replaced the natural background microbiota and established absolute dominance. Following 10 months of long-term frozen storage, the absolute dominance of *Psychrobacter* was disrupted, leading to a substantial decrease in its relative abundance. This decline was accompanied by a significant enrichment of various psychrotolerant and potential spoilage-related taxa, including *Vibrio*, *Psychroserpens*, *Thalassobius*, and *Flavobacterium*. These findings indicate that during long-term frozen storage, the microbial community of mysids undergoes a complete successional trajectory: transitioning from the original environmental microbiota to an outbreak of a single psychrotolerant genus and ultimately shifting towards a more complex microbiota associated with cold tolerance and potential spoilage.

#### 3.3.2. Differential Bacterial Biomarkers (LEfSe)

**Figure 5:**
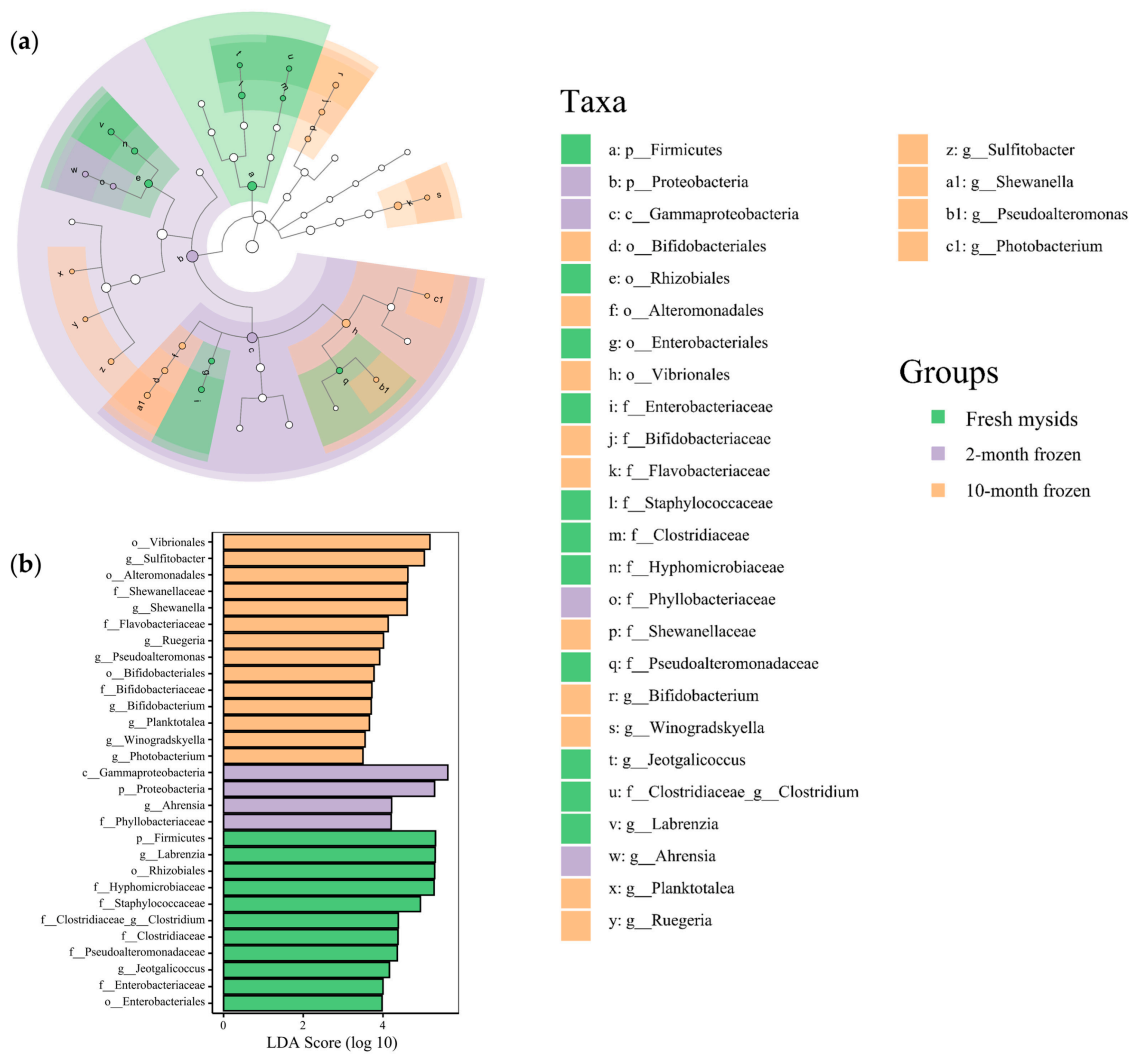
Linear discriminant analysis effect size (LEfSe) identifying differential bacterial biomarkers in mysid shrimp across varying storage durations. (**a**) Cladogram representing the phylogenetic structure and distribution of significantly enriched bacterial taxa among fresh, 2-month frozen, and 10-month frozen groups. (**b**) Bar chart of linear discriminant analysis (LDA) scores showing the differentially abundant bacterial taxa associated with each specific storage condition.

To identify the specific bacterial taxa causing differences in microbial communities across different storage periods, a linear discriminant analysis effect size (LEfSe) was conducted in this study. The phylogenetic cladogram of the microflora (Figure 5a) and the LDA score bar chart (Figure 5b) demonstrated significant differences in the taxonomic structure of the microbiota among the three groups of samples. The significantly enriched biomarkers in the fresh group mainly belonged to the phylum Firmicutes, including the families Staphylococcaceae, Clostridiaceae, and Enterobacteriaceae; at the genus level, *Labrenzia*, *Clostridium*, and *Jeotgalicoccus* were the most discriminative characteristic taxa, representing the unique microflora at the initial stage of storage.

With prolonged storage time, the 2-month frozen group exhibited unique microbial characteristics, with only the phylum Proteobacteria being significantly enriched, centered on the class Gammaproteobacteria; at the genus level, *Ahrensia* (family Phyllobacteriaceae) was the primary differential biomarker for this intermediate group.

In contrast, the 10-month frozen group, representing the long-term freezing treatment, was significantly enriched with a large number of different taxa, most of which are of aquatic environmental origin and associated with food spoilage. The main enriched taxa at the order level in this group included Vibrionales, Alteromonadales, and Bifidobacteriales. Notably, at the genus level, recognized specific spoilage organisms (SSOs) such as *Shewanella*, *Photobacterium*, and *Pseudoalteromonas* were massively enriched, accompanied by a significant increase in the abundance of the genera *Sulfitobacter*, *Ruegeria*, *Bifidobacterium*, *Planktotalea*, and *Winogradskyella*. *Shewanella* and *Photobacterium* emerged as the landmark differential microflora in the 10-month frozen group, directly reflecting the succession patterns of key microflora indicating quality deterioration during long-term frozen storage.

## 4. Discussion

### 4.1. Dynamic Depletion Mechanisms of Nutritional Components Under Prolonged Frozen Storage

The nutritional integrity of frozen mysid shrimp directly determines its efficacy as a staple feed in seahorse aquaculture, influencing their survival, growth, and reproductive performance [4]. Fresh mysids are naturally abundant in pleasant free amino acids (PFAAs), and they are heavily dominated by glycine and aspartate [26]. These specific amino acids function as critical feeding attractants for marine teleosts, stimulating appetite and feed intake. However, after freezing, the concentrations of these essential attractants plummeted by over 50% to 70%. This massive early-stage depletion suggests that conventional freezing procedures instantly trigger intense osmotic stress and cellular lysis, leading to the rapid exudation and enzymatic degradation of water-soluble nutrients, aligning with previously reported protein changes and nutrient losses in frozen aquatic products [8,10]. The rapid loss of these crucial feeding attractants provides a direct biochemical explanation for the poor feeding response and subsequent unstable reproductive performance often observed in captive seahorses fed poorly preserved mysids [27]. Furthermore, while the global FAA profile remained relatively stable between the 2-month and 10-month periods, the continued significant degradation of specific functional amino acids, such as serine, isoleucine, and glutamine at 10 months, indicates that slow but persistent enzymatic hydrolysis is still actively consuming host nutrients even at −20 °C.

It is noteworthy that while the vast majority of nutritional components exhibited a continuous degradation trend, certain metabolic intermediates displayed a unique “V-shaped” fluctuation pattern over the 10-month frozen storage period: an initial significant decline followed by a substantial rebound. Among the free amino acids, citrulline demonstrated the most drastic variation, plummeting from 205.37 μg/g in fresh mysids to a nadir of 9.11 μg/g after 2 months of storage before sharply rebounding to a peak of 289.17 μg/g at 10 months (*p* < 0.05).

Strikingly, this non-linear “V-shaped” trajectory was exceptionally pronounced and widespread across the free fatty acid (FFA) profile. For instance, the content of 13-cis-docosadienoic acid (C22:2) exactly mirrored the citrulline pattern, initially decreasing from 0.41 nmol/g in fresh samples to 0.13 nmol/g at 2 months and then surging to a peak of 0.82 nmol/g at 10 months. Rather than an isolated anomaly, this profound rebound was observed in a broad spectrum of key high-value fatty acids, including arachidonic acid (ARA), myristic acid, palmitoleic acid, and linoleic acid, which all experienced a significant accumulation at 10 months compared to their 2-month nadir. These collective and synchronized fluctuation patterns clearly indicate a highly dynamic and complex process of metabolic remodeling during the late stages of prolonged frozen storage.

Mechanistically, this paradoxical accumulation of FFAs in the late storage phase is a classic biochemical hallmark of severe lipid hydrolysis [7,28]. Initially, this can be attributed to the physical damage induced by the freezing process itself; ice crystal generation and recrystallization during prolonged frozen preservation inevitably break cell membranes and muscle structures, allowing originally compartmentalized endogenous lipases to leak out and accelerate continuous lipid decomposition [29]. However, this endogenous breakdown is severely compounded by exogenous microbial activity during prolonged storage. Our 16S rRNA sequencing results revealed a massive late-stage enrichment of specific spoilage organisms (SSOs), notably *Shewanella* and *Photobacterium* [30]. Although direct in vitro assays of lipase and protease activities were not performed in this study due to experimental constraints, these psychrotrophic bacteria are well-documented to secrete highly active extracellular lipases and proteases [31]. Therefore, it can be strongly inferred that these microbial enzymes synergistically accelerate the decomposition of structural lipids alongside the host’s endogenous enzymes, ultimately driving the massive accumulation of metabolic degradation products.

Consequently, the intense rate of this combined enzymatic lipolysis in severely structurally compromised tissues far exceeds the baseline lipid consumption, resulting in a net accumulation of FFAs [7].

Importantly, these elevated FFA levels at 10 months do not represent nutritional recovery but rather serve as highly unstable substrates that fuel severe secondary degradation. This is directly evidenced by our TBARS analysis, which demonstrated a dramatic spike in peroxide products from 0.225 mg/100 g at 2 months to 1.247 mg/100 g at 10 months (*p* < 0.01). Ultimately, the massive accumulation of these oxidized lipids and decomposed cellular components deeply diminishes the nutritional value of the feed. The ingestion of such structurally degraded and highly oxidized mysids clearly elucidates the biochemical mechanisms driving the elevated mortality and poor gonadal development frequently documented in industrial seahorse aquaculture.

### 4.2. Severe Lipid Peroxidation and Dramatic Accumulation of Protein Spoilage Products During Late-Stage Frozen Storage

The content of thiobarbituric acid reactive substances (TBARSs) in fresh mysids was 0.122 mg/100 g, which only slightly increased to 0.225 mg/100 g after 2 months of storage, with no significant difference between groups; however, when the storage duration was extended to 10 months, this indicator exhibited a dramatic and significant elevation, reaching a maximum of 1.247 mg/100 g. This indicates that the continuous lipid peroxidation and spontaneous autoxidation occurring during prolonged freezing severely deteriorate the feed quality [7]. The massive amount of free fatty acids generated from early-stage lipolysis is highly susceptible to free radical attacks and enzymatic breakdown in a low-temperature environment, ultimately converting into large quantities of toxic secondary oxidation products [19]. The dynamic trend of lipid oxidation is highly consistent with the accumulation pattern of toxic nitrogenous metabolites, both of which surged predominantly in the late storage phase. TVB-N (total volatile basic nitrogen) is the core indicator for evaluating the degree of protein degradation; its content was 0.100 mg/100 g at 2 months of storage, showing no obvious accumulation compared to the fresh samples. By contrast, this substance accumulated massively when stored up to 10 months, peaking at 0.425 mg/100 g. This significant increase proves that as the storage time extends, the dominant spoilage bacteria extensively decompose proteins and amino acids, generating various harmful nitrogenous metabolites.

In striking contrast to the dramatic spikes in TBARS and TVB-N indicators, the histamine concentrations remained consistently stable across all experimental groups, ranging from 8.0875 to 8.7912 μg/mL, with no significant differences among the groups even after 10 months of prolonged frozen storage. This unexpected stability suggests that the conventional −20 °C freezing temperature can effectively inhibit the specific catalytic activity of microbial histidine decarboxylase, blocking the massive conversion of free histidine into histamine under deep-freeze conditions [13]. Furthermore, this result also indicates that although extensive protein decomposition and lipid oxidation occur within the samples at 10 months of storage, the specific biochemical pathway mediating histamine generation and accumulation remains in a suppressed state [32].

The synchronous and sharp elevation of TBARS and TVB-N at 10 months of storage marks a severe deterioration in the safety and quality of the feed. Such harmful substances will reduce the nutritional value of the feed, and the growth performance and reproductive capacity of seahorses will be negatively affected after ingestion [5]. The massive accumulation of these spoilage biomarkers provides a clear toxicological explanation for the high-frequency problems—such as slow growth, reduced spawning, and elevated mortality—that may occur in large-scale seahorse aquaculture when overly stored mysid feed is used. The massive accumulation of these spoilage biomarkers provides a clear toxicological explanation for the high-frequency problems—such as slow growth, reduced spawning, and elevated mortality—that may occur in large-scale seahorse aquaculture when overly stored mysid feed is used. Collectively, these dynamic nutritional depletions and massive accumulations of spoilage biomarkers fully support our initial research hypothesis. It confirms that the nutritional divergence and microecological deterioration caused by prolonged frozen storage act as the core drivers compromising the stable reproductive performance and health of farmed seahorses.

### 4.3. Microecological Mechanisms of Frozen Feed Quality Deterioration Driven by Microbial Community Succession

Fundamentally, the physicochemical and nutritional degradation of frozen feed stems from shifts within its internal microbial community. Our 16S rRNA high-throughput sequencing revealed a distinct, time-dependent successional trajectory within the mysid microbiome during prolonged frozen storage. Initially, the microbial structure of fresh mysids consisted primarily of a natural environmental background flora, dominated by *Synechococcus*. After two months of frozen storage, however, this native flora was almost entirely eradicated. In its place, *Psychrobacter*—a genus highly adapted to low temperatures—rapidly multiplied and established absolute dominance. This shift aligns closely with previous microbiome studies on cold-stored crustaceans, demonstrating that *Psychrobacter* can survive severe freezing stress and outcompete background microbes due to its psychrotolerant nature, ultimately emerging as the dominant survivor [15,33]. This phenomenon confirms that vigorous microbial activity persists even under deep-freeze conditions, acting as an ongoing driver of feed degradation.

As the frozen storage period extended to 10 months, the microecological landscape underwent a second critical transition. The absolute dominance of *Psychrobacter* was disrupted, accompanied by an overall increase in community diversity and a notable enrichment of various psychrotolerant and potentially spoilage-associated taxa, including *Vibrio* and *Flavobacterium* [34]. More importantly, LEfSe analysis pinpointed a massive late-stage outbreak of recognized specific spoilage organisms (SSOs), with *Shewanella*, *Photobacterium*, and *Pseudoalteromonas* emerging as the landmark differential taxa [35]. The emergence of these specific groups strongly matches established marine spoilage models. Numerous prior studies have repeatedly highlighted *Shewanella* and *Photobacterium* as aggressive SSOs responsible for the rapid quality degradation of aquatic products in cold environments [36].

The explosive proliferation of these SSOs establishes a direct and compelling mechanistic link to the severe physicochemical deterioration observed at the 10-month mark. Although direct assays of lipase and protease activities were not performed in the present study due to experimental constraints, these heavily enriched taxa (such as *Shewanella* and *Photobacterium*) are well-documented in the previous literature to secrete highly active extracellular lipases and proteases [31]. Consistent with this, the intense metabolic activities of these dominant SSOs over the 10-month freezing period, as indirectly evidenced by the massive accumulation of their terminal degradation products (FFAs and TVB-N observed in our chemical analysis), strongly suggest that they directly accelerated the large-scale breakdown of structural lipids and host proteins. Consequently, this SSO-driven enzymatic degradation clearly explains the underlying biological cause for the dramatic, synchronous spikes in both TBARS and TVB-N indicators at 10 months. Ultimately, these microecological findings confirm that the unchecked succession and late-stage outbreak of specific spoilage organisms act as the core intrinsic mechanism driving the definitive spoilage and quality loss of long-term frozen mysid feed.

### 4.4. Practical Guidance and Storage Strategy Optimization for the Seahorse Aquaculture Industry

This study initially integrates targeted metabolomics (free amino acids and fatty acids), key physicochemical indicators (TBARS, TVB-N, and histamine), and microbial amplicon sequencing to construct a multidimensional evaluation system for the quality deterioration of frozen mysids. Based on this systematic assessment, the present study provides a crucial scientific basis for feed management in large-scale seahorse aquaculture.

First, given that irreversible and profound lipid peroxidation (a surge in TBARS) and extreme microbial spoilage dominated by specific spoilage organisms (SSOs) (significant enrichment of TVB-N) occurred in mysids frozen for a long-term period (10 months), the ingestion of such deteriorated feed will inevitably pose severe physiological stress and toxicological risks to seahorses. Therefore, in industrial practice, the introduction of long-term frozen mysids should be strictly avoided to curb potential declines in survival rates and impaired reproductive potential. Second, this study reveals that although spoilage indicators (such as TVB-N) remain within a safe baseline during the short-term 2-month frozen storage phase, the core nutrients of the feed (particularly key feeding-attractant amino acids and specific free fatty acids) undergo non-negligible degradation and loss during the initial stages of freezing. Accordingly, this study strongly recommends that when using short-term frozen mysids, enterprises should introduce targeted nutritional compensation. Specifically, thawed feed should be top-coated with key depleted nutrients—such as attractant amino acids (glycine and aspartate) and essential fatty acids (ARA and EPA)—at a dosage of 1.0–1.5% of wet weight (e.g., ~10 mg/g glycine) [37]. To ensure optimal efficiency and prevent leaching, natural binders like gelatin (0.5–1.0% *w*/*w*) must be used during coating [38]. This strategy effectively replenishes the early nutritional void caused by freezing. This will replenish the early nutritional void caused by physical freeze–thaw processes. Establishing science-based feed storage time limits and proactive nutritional fortification mechanisms will be an effective pathway to break through the current bottlenecks of sluggish feeding responses and fluctuating reproductive performance in seahorse aquaculture, thereby providing fundamental nutritional security for the steady and sustainable development of this industry.

However, despite providing a multidimensional and systematic evaluation, this study still presents certain limitations. Regarding the setting of storage durations, this experiment only compared three time points: fresh, 2 months, and 10 months. Due to the large span between the 2-month and 10-month marks, the mid-term quality dynamics (e.g., at 4 and 6 months of frozen storage) were not continuously monitored. Future studies should incorporate denser sampling gradients to precisely identify the critical “inflection point” of rapid quality deterioration in mysids, thereby providing more accurate data support for further refining and optimizing scientific storage duration standards for aquaculture feed. Future studies should incorporate direct in vitro enzymatic assays (e.g., quantifying cold-adapted lipase and protease activities) and transcriptomic analyses to further validate the microbial degradative pathways inferred in this study.

## 5. Conclusions

This study provides a comprehensive, multidimensional assessment of the nutritional depletion and microecological deterioration dynamics of frozen mysid shrimp utilized as dedicated feed in seahorse aquaculture. The results demonstrate that prolonged frozen storage at −20 °C for 10 months triggers severe lipid peroxidation and a massive accumulation of total volatile basic nitrogen (TVB-N). The core driver of this late-stage quality deterioration is the explosive proliferation of psychrotrophic specific spoilage organisms (SSOs) driven by microbial succession, with *Shewanella*, *Photobacterium*, and *Pseudoalteromonas* emerging as the absolute dominant genera. These spoilage bacteria secrete highly active extracellular hydrolytic enzymes that extensively degrade tissue structural lipids, directly causing a paradoxical rebound in free fatty acid content during long-term storage. In contrast, although short-term frozen storage (2 months) effectively suppresses microbial spoilage, the physical damage from freeze-induced cellular lysis leads to the rapid and drastic depletion of key water-soluble feeding attractants, notably glycine and aspartate. In conclusion, this study profoundly elucidates the biochemical and microecological mechanisms underlying the stage-specific deterioration of frozen feeds, establishing a robust nutritional and safety foundation to drive the sustainable development of the commercial seahorse aquaculture industry.

## Figures and Tables

**Table 1:** Free amino acid contents of mysids under different frozen storage periods.

FAA	Fresh Mysids	2-Month Frozen	10-Month Frozen
	μg·g ^−1^		
4-Aminobutyric Acid	16,292.86 ± 2088.63 ^a^	6949.27 ± 1022.08 ^b^	5462.48 ± 550.76 ^b^
Serine	18.11 ± 2.40 ^a^	19.98 ± 3.67 ^a^	5.70 ± 0.52 ^b^
Proline	1379.82 ± 1186.19	493.43 ± 415.09	430.89 ± 367.46
Valine	552.50 ± 69.43 ^a^	17.34 ± 3.64 ^b^	32.50 ± 2.76 ^b^
L-Homoserine	2183.31 ± 232.09 ^a^	898.59 ± 107.96 ^b^	480.70 ± 44.08 ^c^
Threonine	112.40 ± 8.91 ^a^	34.78 ± 6.44 ^b^	47.33 ± 6.84 ^b^
Cysteine	1086.61 ± 138.14 ^a^	384.04 ± 32.24 ^b^	291.69 ± 27.58 ^b^
Taurine	2188.17 ± 255.68 ^a^	722.88 ± 100.75 ^b^	576.50 ± 54.29 ^b^
Leucine	1707.91 ± 216.71 ^a^	559.74 ± 85.57 ^b^	451.79 ± 46.33 ^b^
Hydroxyproline	2.70 ± 0.78 ^a^	0.86 ± 0.15 ^b^	0.58 ± 0.11 ^b^
Isoleucine	266.33 ± 25.63 ^a^	206.95 ± 2.13 ^b^	209.52 ± 20.40 ^b^
Ornithine	1475.06 ± 175.67 ^a^	660.00 ± 44.09 ^b^	520.58 ± 41.05 ^b^
Asparagine	26.51 ± 3.41 ^a^	6.91 ± 1.66 ^b^	7.70 ± 0.81 ^b^
Aspartate	1244.00 ± 143.93 ^a^	438.15 ± 21.69 ^b^	265.30 ± 22.18 ^c^
Homocysteine	2899.63 ± 438.32 ^a^	1879.49 ± 246.86 ^b^	1054.73 ± 92.05 ^c^
S-Methyl-L-Cysteine	347.07 ± 42.28 ^a^	554.03 ± 82.80 ^b^	97.14 ± 3.82 ^c^
Glutamine	5337.56 ± 820.99 ^a^	1427.74 ± 202.44 ^b^	793.48 ± 55.64 ^b^
Lysine	1.09 ± 0.09	N/Q	N/Q
Glutamate	69.88 ± 6.23 ^a^	22.05 ± 4.46 ^b^	9.66 ± 0.83 ^c^
Methionine	412.33 ± 27.10 ^c^	663.60 ± 36.37 ^a^	528.88 ± 52.76 ^b^
3-Hydroxyanthranilic Acid	2103.33 ± 267.58 ^a^	900.41 ± 73.53 ^b^	681.89 ± 52.05 ^b^
Histidine	2054.63 ± 239.35 ^a^	993.45 ± 79.89 ^b^	784.57 ± 78.44 ^b^
Aminoadipic Acid	1230.06 ± 144.74 ^a^	467.39 ± 32.88 ^b^	336.11 ± 28.62 ^b^
5-Hydroxylysine	6.47 ± 0.19 ^a^	0.47 ± 0.13 ^b^	1.08 ± 0.52 ^b^
Phenylalanine	400.68 ± 57.01 ^a^	168.53 ± 27.43 ^b^	137.52 ± 16.18 ^b^
1-Methylhistidine	218.88 ± 25.56 ^a^	36.35 ± 6.65 ^b^	29.36 ± 3.42 ^b^
Arginine	6.91 ± 1.18 ^a^	2.28 ± 0.49 ^b^	1.59 ± 0.20 ^b^
Citrulline	354.66 ± 30.86 ^a^	186.44 ± 8.71 ^b^	143.77 ± 13.09 ^c^
Tyrosine	10.58 ± 0.35 ^a^	6.31 ± 0.99 ^b^	3.68 ± 0.22 ^c^
Tryptophan	62.52 ± 5.91 ^a^	28.00 ± 2.25 ^b^	26.45 ± 2.85 ^b^
Carnosine	205.37 ± 41.61 ^b^	9.11 ± 1.46 ^c^	289.17 ± 34.96 ^a^
Cystine	1461.16 ± 118.70 ^a^	474.10 ± 49.09 ^b^	218.38 ± 11.41 ^c^
Glutathione	377.71 ± 38.08 ^a^	163.50 ± 10.87 ^b^	121.61 ± 9.25 ^b^
4-Aminobutyric Acid	23.19 ± 5.26 ^a^	3.02 ± 0.47 ^b^	1.03 ± 0.38 ^b^
Serine	948.77 ± 259.48 ^a^	231.15 ± 37.40 ^b^	105.26 ± 10.58 ^b^
Proline	0.67 ± 0.21 ^a^	0.21 ± 0.03 ^b^	0.18 ± 0.06 ^b^

Note: Different superscript lowercase letters in the same row indicate significant differences among the different frozen storage periods (*p* < 0.05, Duncan’s post hoc test). N/Q: Detected but below the limit of quantification.

**Table 2:** Free fatty acid contents of mysids under different frozen storage durations.

FFAs	Fresh Mysids	2-Month Frozen	10-Month Frozen
	nmol·g ^−1^		
Hexanoic acid (Caproic, C6)	13.11 ± 4.58	16.07 ± 11.05	11.94 ± 1.92
Octanoic acid (Caprylic, C8)	6.94 ± 1.84	15.63 ± 8.33	9.17 ± 0.48
Nonanoic acid (Pelargonic, C9)	4.66 ± 3.27	9.23 ± 9.0	3.14 ± 1.31
Decanoic acid (Capric, C10)	0.77 ± 0.06 ^b^	0.67 ± 0.18 ^b^	1.29 ± 0.33 ^a^
10-cis-Undecenoic acid (C11:1)	0.09 ± 0.08	0.10 ± 0.01	0.11 ± 0.01
Undecanoic acid (C11)	0.55 ± 0.05 ^a^	0.25 ± 0.05 ^c^	0.42 ± 0.04 ^b^
11-cis-Dodecenoic acid (C12:1)	0.59 ± 0.09 ^a^	0.11 ± 0.00 ^c^	0.22 ± 0.01 ^b^
Dodecanoic acid (Lauric, C12)	3.08 ± 0.74	1.47 ± 1.26	3.67 ± 1.82
12-cis-Tridecenoic acid (C13:1)	N/A	N/A	0.44 ± 0.07
Tridecanoic acid (C13)	6.02 ± 1.18 ^a^	0.67 ± 0.03 ^b^	1.04 ± 0.12 ^b^
9-cis-Tetradecenoic acid (C14:1)	4.95 ± 0.98 ^a^	0.07 ± 0.03 ^b^	0.45 ± 0.06 ^b^
9-trans-Tetradecenoic acid (C14:1)	0.51 ± 0.05 ^a^	0.09 ± 0.01 ^c^	0.23 ± 0.03 ^b^
Tetradecanoic acid (Myristic, C14)	121.76 ± 8.75 ^a^	11.59 ± 0.53 ^c^	27.55 ± 2.25 ^b^
Pentadecanoic acid (C15)	54.14 ± 5.98 ^a^	6.58 ± 0.98 ^b^	10.15 ± 1.19 ^b^
9-cis-Hexadecenoic acid (C16:1)	250.07 ± 29.36 ^a^	16.69 ± 3.08 ^b^	33.56 ± 3.11 ^b^
Hexadecanoic acid (Palmitic, C16)	38.94 ± 0.82 ^a^	36.45 ± 3.64 ^ab^	31.88 ± 1.41 ^b^
10-cis-Heptadecenoic acid (C17:1)	23.22 ± 5.31 ^a^	4.32 ± 1.11 ^b^	5.05 ± 0.57 ^b^
10-trans-Heptadecenoic acid (C17:1)	13.97 ± 3.25 ^a^	1.39 ± 0.33 ^b^	2.26 ± 0.14 ^b^
Heptadecanoic acid (C17)	63.79 ± 2.90 ^a^	16.34 ± 2.98 ^b^	20.20 ± 3.50 ^b^
6-cis,9-cis,12-cis-Octadecatrienoic acid (C18:3)	18.53 ± 3.24 ^a^	0.49 ± 0.02 ^b^	1.66 ± 0.11 ^b^
9-cis,12-cis,15-cis-Octadecatrienoic acid (C18:3)	116.74 ± 10.34 ^a^	6.40 ± 0.79 ^c^	31.22 ± 3.81 ^b^
9-cis,11-trans-Octadecadienoic acid (C18:2)	121.50 ± 14.44 ^a^	17.87 ± 2.41 ^c^	45.29 ± 1.49 ^b^
9-Octadecenoic acid (C18:1)	619.24 ± 40.81 ^a^	335.42 ± 77.32 ^b^	404.11 ± 50.94 ^b^
Octadecanoic acid (Stearic, C18)	37.44 ± 2.96	36.32 ± 2.31	33.83 ± 4.03
10-cis,13-cis-Nonadecadienoic acid (C19:2)	2.47 ± 0.55 ^a^	0.33 ± 0.08 ^b^	0.64 ± 0.07 ^b^
7-cis-Nonadecenoic acid (C19:1)	7.72 ± 1.74 ^a^	0.60 ± 0.13 ^b^	1.24 ± 0.26 ^b^
10-cis-Nonadecenoic acid (C19:1)	25.28 ± 4.24 ^a^	9.22 ± 2.42 ^b^	11.23 ± 2.64 ^b^
Nonadecanoic acid (C19)	31.02 ± 4.78 ^a^	6.88 ± 1.54 ^b^	13.16 ± 2.35 ^b^
5,8,11,14,17-Eicosapentaenoic acid (C20:5)	258.71 ± 5.90 ^a^	173.21 ± 25.00 ^b^	204.74 ± 13.49 ^b^
Arachidonic acid (C20:4)	118.71 ± 5.72 ^a^	26.73 ± 7.18 ^c^	43.95 ± 4.42 ^b^
11-cis,14-cis,17-cis-Eicosatrienoic acid (C20:3)	28.48 ± 4.50 ^a^	9.56 ± 2.27 ^c^	16.94 ± 2.27 ^b^
11-cis,14-cis-Eicosadienoic acid (C20:2)	13.96 ± 2.10	11.06 ± 2.67	13.07 ± 2.04
11-cis-Eicosenoic acid (C20:1)	39.18 ± 6.35 ^a^	25.51 ± 6.27 ^b^	39.67 ± 5.67 ^a^
8-cis-Eicosenoic acid (C20:1)	15.04 ± 1.20 ^b^	8.38 ± 2.92 ^c^	22.69 ± 2.85 ^a^
Eicosanoic acid (Arachidic, C20)	35.32 ± 5.83 ^a^	17.95 ± 2.77 ^b^	26.03 ± 4.32 ^b^
12-cis,15-cis-Heneicosadienoic acid (C21:2)	1.46 ± 0.24 ^a^	N/A	0.18 ± 0.01 ^b^
14-cis-Tricosenoic acid (C23:1)	3.99 ± 0.39 ^a^	2.00 ± 0.57 ^c^	2.95 ± 0.16 ^b^
12-cis-Heneicosenoic acid (C21:1)	0.93 ± 0.12 ^a^	0.28 ± 0.04 ^c^	0.74 ± 0.09 ^b^
Heneicosylic acid (C21)	7.25 ± 1.33 ^a^	1.91 ± 0.39 ^b^	2.48 ± 0.61 ^b^
4,7,10,13,16,19-Docosahexaenoic acid (C22:6)	100.43 ± 13.36	98.48 ± 16.87	106.33 ± 3.63
4,7,10,13,16-Docosapentaenoic acid (C22:5)	25.41 ± 2.53 ^a^	1.99 ± 0.31 ^b^	3.30 ± 0.26 ^b^
7,10,13,16,19-Docosapentaenoic acid (C22:5)	6.93 ± 0.49 ^a^	1.22 ± 0.14 ^c^	5.01 ± 0.67 ^b^
7,10,13,16-Docosatetraenoic acid (C22:4)	1.88 ± 0.21 ^a^	0.14 ± 0.01 ^c^	0.47 ± 0.01 ^b^
13-cis,16-cis,19-cis-Docosatrienoic acid (C22:3)	0.31 ± 0.03 ^a^	0.04 ± 0.00 ^b^	0.29 ± 0.01 ^a^
13-cis,16-cis-Docosadienoic acid (C22:2)	0.41 ± 0.04 ^b^	0.13 ± 0.02 ^c^	0.82 ± 0.15 ^a^
13-cis-Docosenoic acid (C22:1)	3.75 ± 0.02 ^a^	2.69 ± 0.60 ^b^	3.52 ± 0.29 ^a^
Docosanoic acid (Behenic, C22)	12.90 ± 0.86 ^a^	3.58 ± 0.61 ^b^	4.70 ± 1.12 ^b^
Tricosanoic acid (C23)	3.89 ± 0.26 ^a^	0.73 ± 0.13 ^b^	0.72 ± 0.16 ^b^
15-cis-Tetracosenoic acid (C24:1)	5.16 ± 0.36	4.24 ± 0.89	4.30 ± 0.76
Tetracosanoic acid (Lignoceric, C24)	4.10 ± 0.38 ^a^	0.94 ± 0.36 ^b^	1.62 ± 0.35 ^b^
Hexacosanoic acid (Cerolic acid, C26)	0.81 ± 0.08 ^a^	0.12 ± 0.02 ^b^	0.13 ± 0.01 ^b^

Note: Different superscript lowercase letters in the same row indicate significant differences among the different frozen storage periods (*p* < 0.05, Duncan’s post hoc test). N/A: Not detected.

## Data Availability

The data presented in this study are openly available in NCBI (https://www.ncbi.nlm.nih.gov/bioproject?term=PRJNA1475597&cmd=DetailsSearch (accessed on 8 July 2026)) (PRJNA1475597).
